# Well-being grants in an academic medical center: A case example

**DOI:** 10.1017/cts.2023.615

**Published:** 2023-09-13

**Authors:** Lauren E. Olson, Miriam A. Bredella, Anne S. Levy, Darshan H. Mehta

**Affiliations:** 1 Center for Faculty Development, Massachusetts General Hospital, Boston, MA, USA; 2 NYU Langone Health, New York, NY, USA; 3 Harvard Medical School, Boston, MA, USA

**Keywords:** Well-being, pilot grant, burnout, stress reduction, engagement, social connection, self-care

## Abstract

Academic medical centers (AMCs) rely on engaged and motivated faculty for their success. Significant burnout among clinical and research faculty has resulted in career disengagement and turnover. As such, AMCs must be vested in cultivating faculty engagement and well-being through novel initiatives that support faculty. The Well-Being Education Grants program was established by the Office for Well-Being within the Center for Faculty Development at Massachusetts General Hospital to provide the impetus many faculty needed to dedicate time to their well-being, demonstrating that investments in multi-component interventions around faculty well-being require resources and funding.

## Introduction

Institutions within academic medicine rely on engaged and motivated faculty for their success [1]. However, stress and other factors causing professional burnout [1] among clinical and research faculty can catalyze career disengagement and turnover [2]. For example, physicians with burnout are more than three times more likely to harbor thoughts of leaving or intentions to leave their job than physicians with no burnout who are happy and satisfied with their career choice [2]. Those physicians who work in academic settings can have even higher rates of career turnover compared with physicians at non-academic institutions – up to 38% will leave academia within ten years [3]. Furthermore, compared with mid-career physicians in an academic setting (11–20 years since training), early-career physicians (≤10 years since training) have an increased association with burnout [4]. For research faculty, the necessity of pursuing grants, publication pressure, and a pervasive culture of overwork are significant predictors of burnout [5]. The risk of burnout among younger-aged researchers and physicians is also comparatively higher than among those more senior [5,6]. Women are more likely to experience burnout than men due to competing demands in early career such as parenting and domestic duties [7]. Finally, the pressures to perform are often more intense among underrepresented groups in academic medicine, such as women, people of color, and marginalized communities, leading to a higher risk of burnout [5,8].

Given the link between a lack of career satisfaction and burnout, academic medical institutions must be vested in cultivating faculty engagement and well-being, as improvement of faculty engagement and well-being results in excellent organizational health. Therefore, faculty engagement and well-being should receive the same level of concern as other priorities, such as patient care and financial viability [9]. Engagement is widely considered a positive, fulfilling, work-related state of mind characterized by vigor, dedication, and absorption [10]. Well-being refers to a person’s quality of life and ability to contribute to the world with meaning and purpose [11]. Enthusiastic faculty are more intellectually engaged and will grow personally and professionally throughout their academic careers, continually acquiring new skills and knowledge [1]. It is essential to recognize that efforts to increase engagement cannot fall solely to the individual – such increased engagement must be the shared responsibility of the faculty and the organization [12]. Leadership must provide recognition, show appreciation, and promote faculty self-esteem to support and maintain faculty vitality [3].

More engaged employees feel an energetic and effective connection with their work activities [10]. People who experience less stress and feel more involved with their work also experience increased productivity and creativity, which are keys to success in academic medicine [13]. A range of effective interventions for reducing burnout and improving engagement are available, including those focused on organizational culture, those supporting physicians at the individual level through organizational-funded initiatives, and multicomponent interventions [2]. The Office for Well-Being within the Center for Faculty Development at Massachusetts General Hospital (MGH) focuses on engagement and well-being across the career span by designing initiatives to improve resilience and create a positive work culture that organically integrates well-being.

One of the initiatives to support academic faculty and trainees around resilience and well-being created by the Office for Well-Being is the Well-Being Education Grants program to encourage trainees and faculty at MGH at the individual level to pursue training or continuing education in areas that the individual feels will cultivate their resilience and well-being.

## Methods

The first round of well-being education grants was launched in 2021. The grants were intended to support the resilience and well-being of faculty and trainees through programs and activities with a cap of $750 per grant. Grants are paid with hospital funds allotted to the Center for Faculty Development to disperse based on programmatic priorities. A call for applications was sent to all MGH faculty and trainees (graduate students, post-doctoral fellows, residents, and fellows), including clinicians, educators, and investigators. The call for applications was initially issued three times per year and covered activities over four months; in 2022, the program transitioned to calls twice per year that covered activities over six months. The simple application asked candidates to briefly explain their requested well-being activity and benefit the individual’s sense of resilience or well-being. Awardees were selected by a committee within two weeks after submission based on the strength of their argument that their chosen activity was related to well-being and sufficient information on its nature, location, and cost. As further cycles were launched, certain applicants were given preference: those who had yet to receive funding, those who chose a new activity to pursue rather than an established hobby or pursuit, and those who were more junior in their careers.

A brief post-survey was emailed to grant recipients at the end of each activity cycle. It asked respondents to rate their agreement on a 5-point Likert scale with the following statements: “The Well-Being Education Grant had an impact on my well-being” and “The Well-Being Education Grant contributed to my work-life balance.” Respondents were also asked to answer the qualitative question, “How did the well-being grant impact you?”

## Results

**Figure 1.**
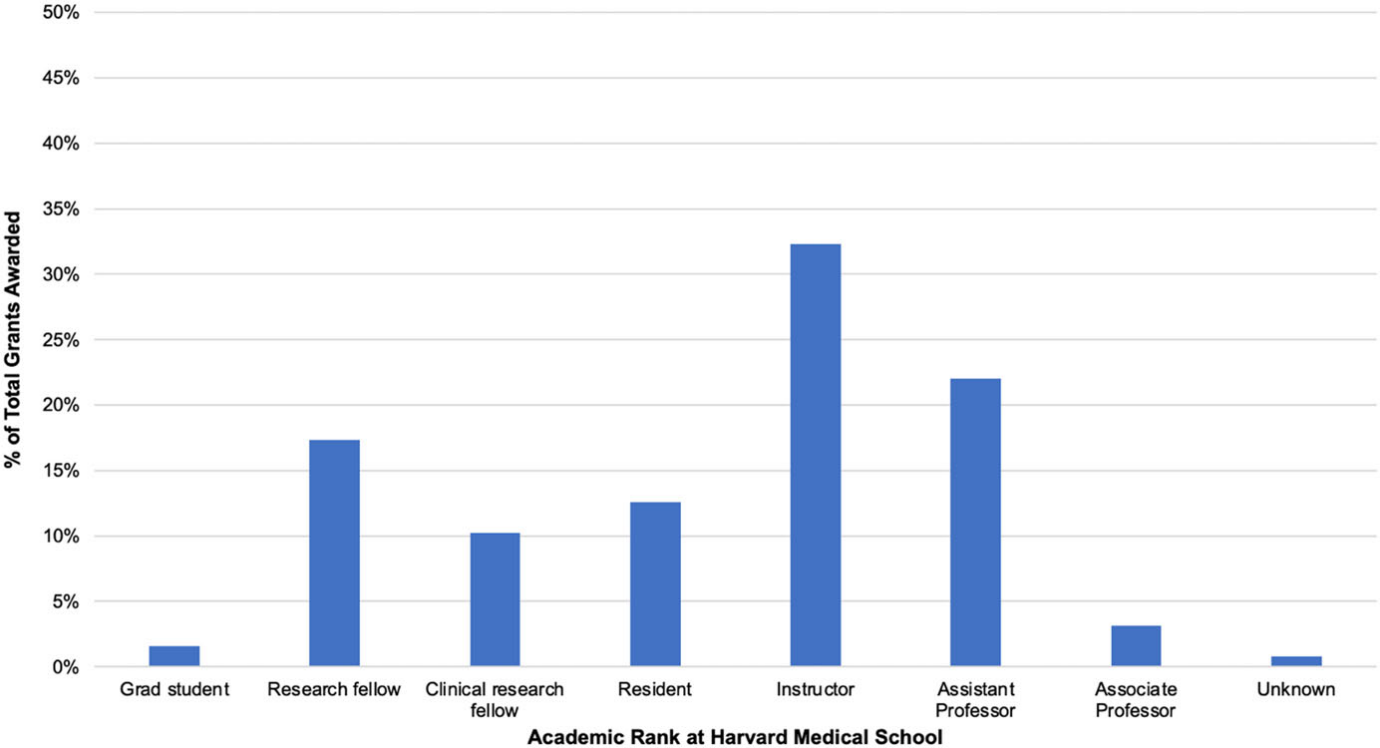
Distribution of grants by academic rank shows the percentage of total grants awarded (n = 128) by Harvard Medical School academic rank.

From February 2021 to February 2023, 268 applications were submitted for five grant cycles. We awarded a total of 128 grants, the total cost coming to $63,543. Examples of awarded activities include stress reduction courses, mindfulness retreats, professional well-being coaching, and various art and fitness classes. Over 73% of those awarded are women. Fig. 1 shows the distribution of grants awarded by Harvard Medical School academic rank. Over these five cycles, the average grant awarded was $496, with a standard deviation of $201.

**Figure 2.**
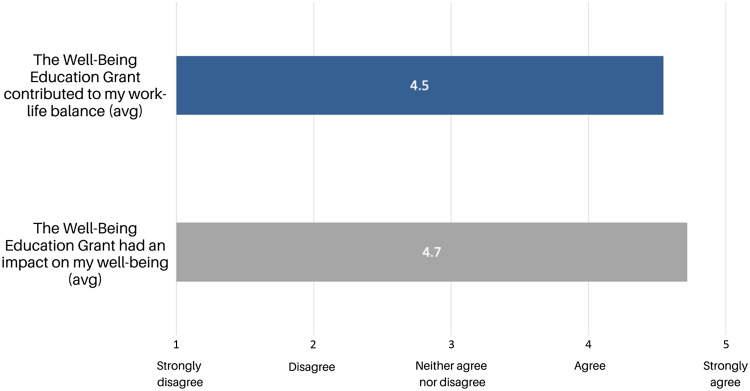
Quantitative results from the feedback survey. Grant recipients were asked to rate their agreement with the two questions in Fig. 2 on a 5-point Likert scale with one point for “strongly disagree” and five points for “strongly agree.” Fig. 2 shows the average from the collected responses.

Approximately 44% of grant recipients responded to the post-survey, and all those who responded gave comments. As shown in Fig. 2, grant recipients agreed that the Well-Being Education Grant contributed to their work-life balance and positively impacted their well-being. As shown in Table 1, three major themes emerged in the qualitative responses collected from grant recipients: stress reduction, well-being/self-care, and social connection. Some respondents felt that the Well-Being Education Grants demonstrate an institutional commitment to the well-being of its employees. Comments included “It felt like a good faith effort by my institution to support me as an employee” and “I felt the MGH valued the importance of life outside work.”

**Table 1.** Qualitative results from the feedback survey. Grant recipients were asked the following qualitative question in a survey: “How did the Well-Being Education Grant impact you?”

Stress Reduction
The educational course that I used the grant was for conflict resolution and management. Conflicts are inherent part of our lives, daily lives and learning to deal and manage them well significantly lessened my stress.
The Well-Being Grant provided support for me to explore meditation through a formal course. This course helped me prioritize mind-body wellness during times of anxiety.
One on-one coaching greatly improved my sense of perspective, reduced stress, and boosted productivity
Provided an opportunity to learn something new and a dedicated time to rest/relax/recharge
Provided me with mindfulness tools to feel more grounded and less stressed
I signed up for an app-based coaching program that integrated well with my busy schedule. It taught me exercises (as short as two minutes) that helped me manage my burnout.
Through the class that I was supported by the grant, I could relieve stress and tension. It was very helpful for finding peace of mind and energy in daily life.
decreased acute stress, learned strategies to manage stress effectively moving forward, made me feel like MGH values my work/life balance and invests in my well being
I had a concentrated experience of wellness and yoga in a warm and beautiful setting. I feel renewed, refreshed and restored.
The Well-Being Grant afforded me an opportunity to try a new kind of art activity (glassblowing), which I otherwise would not have been able to. This was an incredible way to completely step away from my research work and concentrate on a very physical activity that required intense focus and concentration. This was particularly helpful given that I was in the process of applying for some funding, which was rather stressful, so the mental break was much needed.
I have started some of these practices and intend to incorporate them into my routine. I have already noticed an impact in handling stress at work and home.
**Well-Being/Self Care**
Allowed me to pursue music lessons which greatly impacted my day to day joy and wellness
It had a very positive effect on my well-being. I enjoy the funded activity so much; it energizes me, and I also get to make new friends.
Helped set aside time for self-care
It provided an opportunity to consider self-care and develop those skills. It also felt like a good faith effort by my institution to support me as an employee.
Provided access to an online course that focused on tools for growing personal resiliency. I have ongoing access to the materials so can continue to benefit from it. Appreciated that I was able to choose something that spoke to me, i.e., the grants allow a tailored support
Improved sleep
It provided me with a dedicated time and space to focus on a mindful activity that otherwise always falls to the bottom of my priorities.
This was a fantastic opportunity that I am incredibly grateful for. I had been struggling during the COVID pandemic and the Well-Being Education Grant gave me the opportunity to pursue a passion of mine and provided me with coping skills that I will carry through the rest of my training. Thank you.
It covered the costs of taking music lessons, which was a great scheduled reprieve from work and a chance to refresh.
**Social Connection**
The grant supported enrolling in music production lessons, with several benefits. It relieved significant financial stress in pursuing extracurricular; it led to new social experiences; and it shifted the ongoing tenor of downtime towards creative - rather than consumptive- activities.
It allowed me to explore a hobby of mine I otherwise would not have been able to. I look back fondly at the opportunity to connect with people during the watercolor painting class especially during these last few weeks when I have been so busy clinically.
I had the chance to reconnect with my love for music through the grant, I was able to meet new people who shared similar interests and passions, which helped me foster new connections and establish a wider social circle. It also helped me enrich my knowledge of music, and I learned several different techniques to enhance my growth as an amateur vocalist.
It was a fantastic opportunity to be in community with others and reflect.
It has been so, so great to practice yoga in person with other people, after 3 years of practicing in front of a screen. Thank you.
Doing the yoga classes in person also connected me with others and instilled a sense of community, which I had been missing since the pandemic. I am grateful to have had the chance to focus on both my physical and mental health thanks to this grant!

## Discussion

To our knowledge, this is the first initiative in an academic health institution to offer funds at the individual level to support well-being activities for clinical and non-clinical faculty and trainees. These Well-Being Education Grants have reduced stress, improved self-care, and increased faculty and trainees’ social connection. Our faculty and trainees’ appreciation for social connection as beneficial to combat feelings of social distress is consistent with academic researchers [14,15]. Feedback has made clear that this grant provided the impetus many faculty and trainees needed to dedicate a portion of their limited time to their well-being. Although the amount of the award was relatively small, especially from the faculty perspective, the positive responses from awardees suggest they may see this as a gift in appreciation for their immense commitment to their work. Awardees appreciated this grant’s tailored support, as they could choose which activity would be most beneficial and fit best into their life. Many used the funds to reconnect with an abandoned passion or hobby or to focus on practices that reduce stress and increase mindfulness. Of the 128 total grant recipients over five grant cycles, 20% chose to pursue some form of mindfulness, resilience-building, or stress reduction course. This choice is consistent with one study among doctoral students, which found that they desired more proactive support measures, such as workshops designed to build individual resilience and mindfulness training [15]. Fifty-eight percent of grant recipients used the funds for live group activities, whether in-person or online, such as narrative medicine courses or yoga retreats. The other 42% of recipients used the funds for asynchronous individual activities such as personal coaching or voice lessons. All chosen activities use institutional funds to give faculty and trainees time away from the clinic or the lab, which is essential for stress reduction [13]. Using institutional funds to offer the opportunity to take time away from work demonstrates to employees the value the institution places on their work-life balance. A limitation of this study that should be considered is that applicants who did not receive a grant were not surveyed. Future examinations should examine the effect on those not receiving a grant.

These grants serve a dual purpose in Massachusetts. In 2019, the Massachusetts Board of Registration in Medicine approved a policy expanding Risk Management CMEs to include wellness and burnout prevention [16]. This policy allows physicians to fulfill seven of the ten required hours of their Risk Management credits with well-being topics [16]. Some grant recipients chose to pursue well-being activities that satisfy their required Risk Management hours.

While this article focuses on the feasibility of the Well-Being Education Grants program, this program is part of a multi-modal approach to well-being. For example, our Office for Well-Being has instituted other opportunities to support well-being at the individual level, including twice-weekly meditations, creative activities every two weeks (“Fun Friday”), TEDxMGH talks, and small group wellness coaching [17]. The various opportunities offered by the Office for Well-Being provide unexpected and spontaneous connection and the comforting and energizing recognition of shared beliefs, challenges, and interests. Coming together, whether for meditation, skills-building, or storytelling, catalyzes moments of creative epiphany. Multi-component interventions ensure that MGH improves employee wellness from various angles to reach as many people as possible based on their preferences. Many of our programs and resources are offered virtually or on our website to ensure they are accessible to all, such as busy trainees. Online sources are crucial for those more junior in academic medicine, such as postdocs, seeking non-academic support [15]. Offerings may also require targeted approaches to populations with specific needs. For example, to address the risk of burnout among new parents at MGH [18], the Parental Wellness Program provides feeding and lactation stipends and a paired parent mentor.

Well-being interventions do require investment from the organization in administrative support. Organizational leadership must know about these opportunities and understand their value for employees’ well-being. While it is true that these interventions require resources and funding (beyond the time and energy investment), such resources and funding are not nearly as onerous as one might think. We have found that employees most appreciate that the institution cares about them and their well-being. While the amount of investment requires further study, we found that even a modest investment has the potential for significant impact. The potential return on an institution’s investment in well-being is increased engagement and enthusiasm among faculty and gratitude toward institutional leadership for cultivating a culture of care for its employees. Faculty who feel engaged with their careers and satisfied with institutional support tend to be more loyal to their place of work, another significant potential return on investment.

The National Academy of Medicine released the National Plan for Health Workforce Well-Being in October 2022, “intended to inspire collective action that focuses on changes needed across the health system and at the organizational level to improve the well-being of the health workforce” [19]. Two of the seven priority areas for this plan are to address policy barriers for daily work and to institutionalize well-being as a long-term value [19]. Organizationally funded initiatives such as the Well-Being Education Grants are one step toward shifting policy to support the well-being of the health workforce in academic medical institutions. Future studies should look at the effect of this more systematically, recognizing that using a randomization trial in this type of work would not be appropriate, given that well-being programs should be available to all.
